# Exploration of acute gout diagnosis based on ultrasound viscoelastic imaging: quantitative parameter analysis and clinical validation

**DOI:** 10.3389/fmed.2025.1729517

**Published:** 2025-12-17

**Authors:** Jiesi Zhang, Xiao Chen, Peipei Li, Mei Zhao, Li Li, Yuquan Ye

**Affiliations:** 1 Department of Ultrasound Medicine, Hebei Medical University, Shijiazhuang, China; 2 Department of Ultrasound, Hebei General Hospital, Shijiazhuang, China

**Keywords:** acute gout, ultrasound imaging, viscosity, elasticity, diagnostic efficacy

## Abstract

**Objectives:**

To explore the optimal quantitative parameters and clinical application value of ultrasound viscoelastic imaging in the differential diagnosis of acute gout (AG) and non-AG.

**Methods:**

This study enrolled 102 patients presenting with acute joint swelling and pain, and divided them into the AG group and non-AG group. Differences in viscoelastic quantitative parameters, including the shear wave velocity (C), viscosity coefficient (V) and dispersion coefficient (D), between the two groups were compared. Parameters with significant differences were included in generalized estimating equations (GEE) analysis to screen out the optimal parameters for predicting AG, and receiver operating characteristic (ROC) curves were plotted to evaluate the diagnostic efficacy of each parameter alone and in combination.

**Results:**

The AG group included 45 patients, and the non-AG group included 57 patients. There were statistically significant differences in C_mean_, C_max_, C_min_, C_SD_, V_mean_, V_max_, V_SD_, D_mean_, D_max_, and D_SD_ between the two groups (all *P* < 0.05). GEE analysis showed that the parameters finally included in the equation were C_mean_ and D_max_, and the equation was Logit(P) = (−11.186) + (3.456 × C_mean_) + (0.191 × D_max_). ROC curve analysis revealed that among single parameters, C_mean_ had the highest AUC of 0.836 (95% CI 0.774∼0.897), with an optimal cutoff value of 2.36 m/s. The AUC of the GEE model was 0.885 (95% CI 0.833∼0.938), with an optimal cutoff value of 0.41. The AUC of the GEE model combining multiple parameters was superior to that of single-parameter diagnosis (*P* = 0.033).

**Conclusion:**

Among ultrasound viscoelastic parameters, C_mean_ and D_max_ are effective non-invasive quantitative indicators for diagnosing AG, and the diagnostic efficacy of combined multi-parameter diagnosis is superior to that of single-parameter diagnosis.

## 1 Introduction

Gout is a crystal-related arthropathy caused by the deposition of monosodium urate (MSU), which is directly associated with hyperuricemia resulting from purine metabolism disorders and/or reduced uric acid excretions. With the progression of the disease, inflammation may lead to permanent damage and destruction of articular cartilage and bone. Without effective and timely treatment, gout can result in adverse outcomes such as disability, loss of work capacity, and loss of self-care ability. With the development of social economy and changes in people’s dietary structure, the prevalence of gout has been increasing year by year and tends to be younger. The global prevalence of gout ranges from 0.1% to 6.8% (1).

Currently, the internationally recognized gold standard for the diagnosis of gout is the detection of urate crystals via joint aspiration (2). However, for patients with a small amount of joint effusion or involvement of small joints, joint aspiration is technically challenging (3). Additionally, needle aspiration biopsy is an invasive procedure with potential risks of complications. Therefore, it is crucial to explore a non-invasive and reliable diagnostic method. In recent years, imaging techniques have made significant progress in the diagnosis and evaluation of gout. The 2015 gout classification criteria issued by the American College of Rheumatology and the European League Against Rheumatism (ACR/EULAR) incorporated ultrasound and dual-energy computed tomography (DECT) as new effective tools for gout diagnosis (4). High-frequency ultrasound is cost-effective and convenient, allowing real-time dynamic observation of lesions, which makes it suitable for routine screening and efficacy follow-up. Specific ultrasound signs (double contour sign, tophus) have high specificity and high positive predictive value for early gout, but their sensitivity is relatively limited (5, 6). Therefore, exploring a new non-invasive, simple technique that can improve the sensitivity of ultrasound diagnosis has become the direction of our research.

Shear wave elastography (SWE) is a non-invasive dynamic imaging technique capable of quantifying changes in tissue elasticity (stiffness). It reflects differences in tissue elasticity by measuring the propagation velocity of directional shear waves emitted by ultrasonic pulses, thereby acquiring quantitative information regarding the elasticity of biological tissues. However, current SWE techniques treat tissues as homogeneous pure elastic bodies, while living tissues are heterogeneous and complex biomechanical structures that exhibit both elastic solid properties and viscous fluid properties. Owing to the viscous nature of biological tissues, the propagation velocity of shear waves within them varies with frequency, a phenomenon known as dispersion during propagation (7). Based on conventional shear wave elastography, ultrasound viscoelastic imaging incorporates the dispersion factor. Utilizing a novel rheological model (the VOIGT model) (8), it quantifies the differences in shear wave velocities at different frequencies to calculate two new quantitative parameters: the viscosity coefficient (V) and the dispersion coefficient (D), which can reflect the viscous characteristics of tissues. Simultaneously, it can also obtain the shear wave velocity(C) and elastic modulus (E), which are capable of reflecting the elastic (stiffness) characteristics of tissues(9).Ultrasound viscoelastic imaging is an emerging technique that has garnered increasing attention in recent years. It has demonstrated several advantages in the assessment of liver fibrosis and liver grafts (10–12), non-invasive evaluation of chronic kidney disease (13), and differentiation of benign and malignant breast lesions (14–16). Currently, there is a paucity of studies on the application of viscoelastography in gout. This study aimed to explore the application value of multiple ultrasonic shear wave viscoelastic parameters in the diagnosis of acute gout and identify the optimal quantitative parameters.

## 2 Materials and methods

### 2.1 Patients

This retrospective study analyzed 102 outpatients and inpatients (involving 151 joints) who presented with joint swelling and pain at a single center from April 2024 to March 2025. According to the corresponding clinical guidelines, the patients were divided into the acute gout (AG) group and the non-AG group.

Inclusion criteria: (1) All patients had acute onset of joint swelling and pain involving ≥ 1 joint within 3 days before ultrasound examination; this could be either the first episode of joint swelling and pain or an acute exacerbation of chronic joint disease. (2) All patients underwent grayscale ultrasound and ultrasonic shear wave viscoelastography examinations. (3) Grayscale ultrasound showed synovial hypertrophy in the swollen and painful joints.

Exclusion criteria: (1) Pregnant or lactating women; (2) Patients with more than two concurrent joint diseases; (3) Patients who had taken uric acid-lowering drugs or hormonal therapeutic drugs within the past one month; (4) Patients with complicated malignant tumors; (5) Patients with cognitive or intellectual impairment and poor clinical compliance.

**FIGURE 1:**
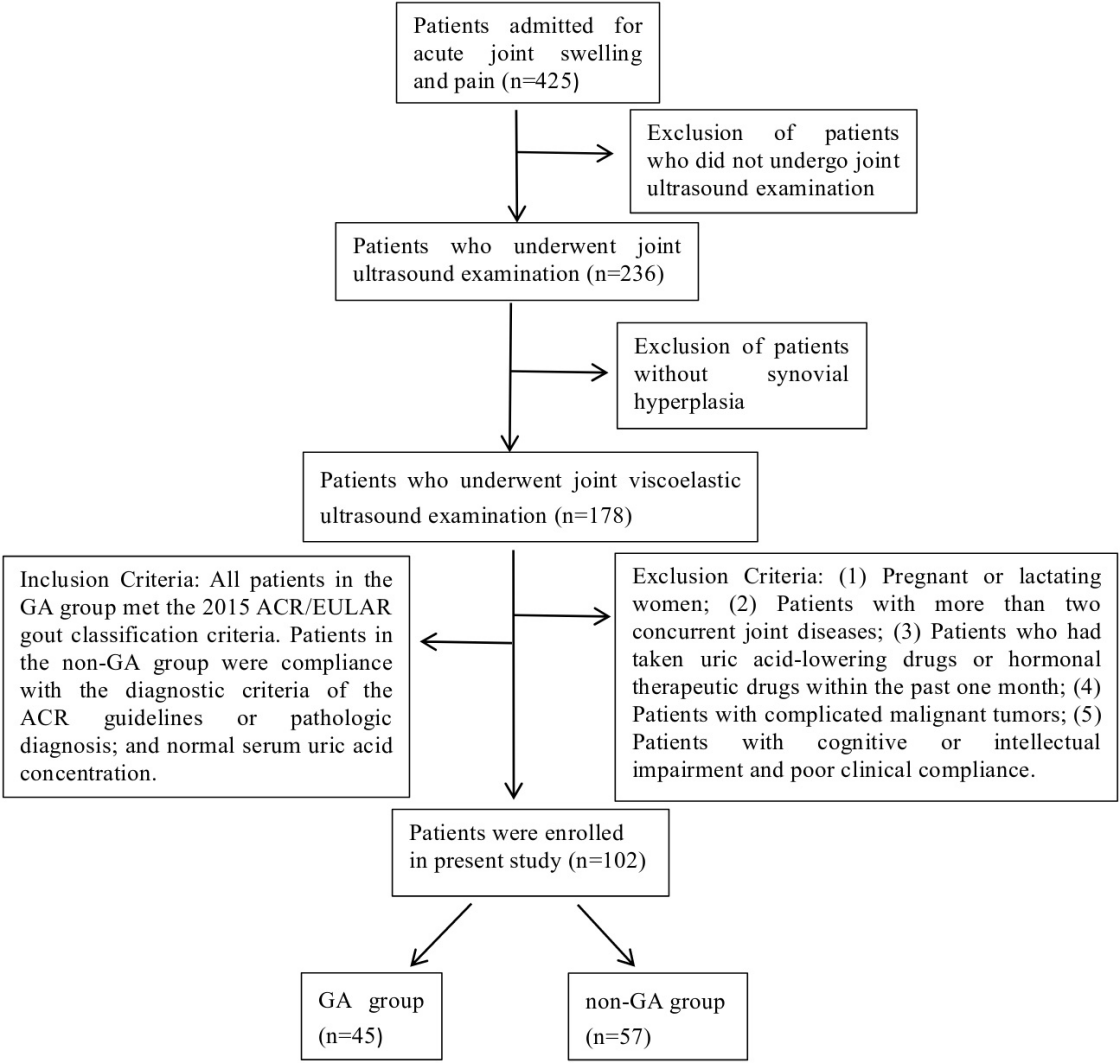
Flowchart of patient selection in this study.

Grouping criteria: All patients in the acute gout (AG) group met the 2015 American College of Rheumatology/European League Against Rheumatism (ACR/EULAR) gout classification criteria (4). Patients in the non-AG group were compliance with the diagnostic criteria of the American College of Rheumatology guidelines or pathologic diagnosis; and normal serum uric acid concentration. All patients were independently diagnosed by two physicians with more than 10 years of clinical experience in rheumatology and immunology. Patients in the AG group were strictly diagnosed in accordance with the 2015 ACR/EULAR gout classification criteria, while those in the non-AG group were diagnosed based on the ACR diagnostic criteria for the corresponding disease or pathological diagnosis results. For patients with uncertain diagnosis (e.g., atypical clinical manifestations, ambiguous laboratory indicators), their grouping was collectively determined through departmental case discussions. Patients with two or more concurrent joint diseases were excluded in accordance with the exclusion criteria (a total of 3 such patients were excluded), as concurrent diseases might interfere with the analysis of viscoelastic parameters (Figure 1).

This study was conducted in accordance with the Declaration of Helsinki and was approved by the Ethics Committee of Hebei General Hospital (No. 2025-247), and registered at the China Clinical Trial Registration Centre (ChiCTR2500103688). All ultrasound examinations were performed as part of routine clinical practice, with prior informed consent obtained from patients or their legal guardians.

### 2.2 Sample size calculation

The sample size of this study was estimated based on pilot study data (*n* = 20). Taking the core viscoelastic parameter C_mean_ (mean shear wave velocity) as the primary outcome measure, the effect size was set to 0.8, the significance level α = 0.05 (two-tailed), and the power (1−β) = 80%. Calculations were performed using G*Power 3.1 software, and the results showed that the minimum required total sample size was 84 cases. A total of 102 patients were finally included in this study, which exceeded the minimum sample size requirement and ensured sufficient statistical power for the comparison of key parameters.

### 2.3 Instrumentation

A Mindray Resona A20 diagnostic ultrasound system (Shenzhen, China) equipped with a linear array transducer (frequency range: 3–15 MHz) was used.

### 2.4 Ultrasound examinations

Gray-scale ultrasound and viscoelastic ultrasound examinations were performed on all subjects by the same ultrasonographer. Before the examination, patients were in a resting state, and their positions were adjusted according to the examination site with the affected joints fully exposed. The ultrasound examination method for each joint was conducted with reference to the ESSR Musculoskeletal Ultrasound: Technical Guidelines (17). Grayscale scanning was performed on the swollen and painful joints, with observation and documentation of synovial thickness, joint effusion, bone erosion, double contour sign, and tophus.

**FIGURE 2:**
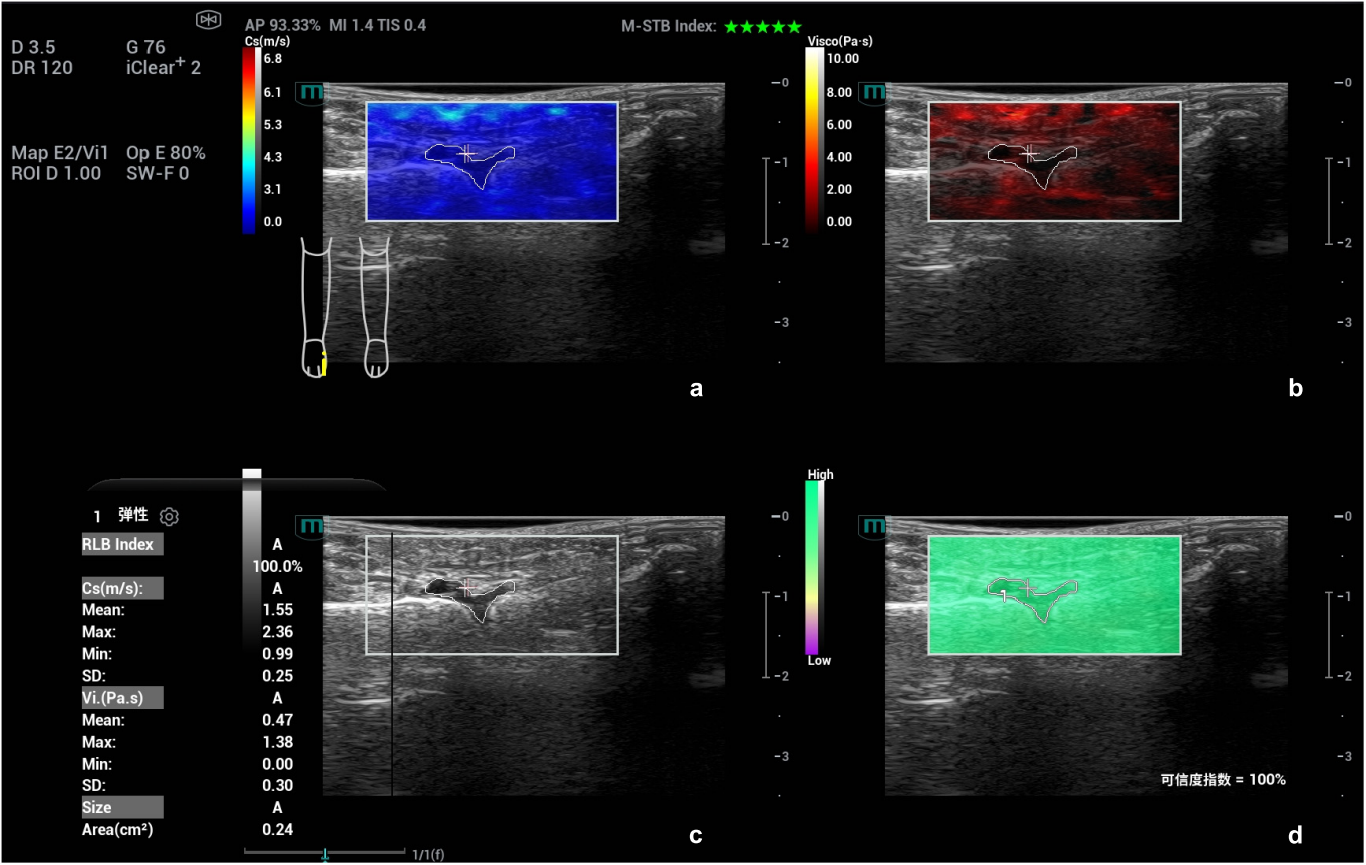
Schematic diagram of viscoelastic images (measurement of shear wave velocity and viscosity coefficient in the first metatarsophalangeal joint of the right foot). **(a)** (top left) Shear wave elasticity image; **(b)** (top right) Viscosity coefficient image; **(c)** (bottom left) Grayscale ultrasound image; **(d)** (bottom right) Reliability map (RLB).

Subsequently, based on gray-scale ultrasound, the maximum longitudinal section of synovial thickening in the joint was obtained for ultrasonic shear wave viscoelastography examination. When acquiring images, the probe was gently placed in contact with the patient’s skin, and stabilized with minimal pressure. The acquisition frame covered the entire lesion area. To reduce interference from respiratory movement, patients were instructed to remain calm and hold their breath for 3–5 s. Then, the viscoelastic examination was activated to enter the four-panel mode, which simultaneously displayed the gray-scale image, shear wave elasticity images, viscosity images and reliability map (RLB) (Figure 2). The system integrated quality control modes including motion stability index (M-STB) and RLB to improve data stability and reliability. M-STB was graded on a scale of 1–5 stars, which dynamically evaluated motion interference between the biomedical sensor and the subject’s skin. Images were considered to be acquired under stable conditions when M-STB ≥ 4 stars. The RLB displayed areas with high reliability in green and areas with low reliability in purple. The RLB index indicated the reliability of the image as a percentage, and images were deemed qualified when the RLB index > 90%. All physicians performing ultrasound examinations and parameter analysis were completely blinded to the patients’ clinical information and did not refer to any clinical data during the operation, including demographic characteristics, medical history, laboratory results, joint aspiration findings, clinical diagnoses, and treatment plans.

### 2.5 Image analysis

Synovial hypertrophy was assessed on single grayscale images, primarily characterized by hypoechoic areas within the joint. In this study, the definition of synovial thickening refers to the EULAR-OMERACT semi-quantitative scoring system(18), Grade 0 = No synovial hypertrophy (SH) independently of the presence of effusion; Grade 1 = Minimal hypoechoic SH up to the level of the horizontal line connecting bone surfaces between the metacarpal head and the proximal phalanx; Grade 2 = Moderate hypoechoic SH extending beyond joint line but with the upper surface concave (curved downwards) or hypertrophy extending beyond the joint line but with the upper surface flat; Grade 3 = Severe hypoechoic SH with or without effusion extending beyond the joint line but with the upper surface convex (curved upward). Patients with a synovial grade of 1–3 are considered to have synovial hypertrophy and included in this study, while those with Grade 0 (no synovial thickening) are excluded.

**FIGURE 3:**
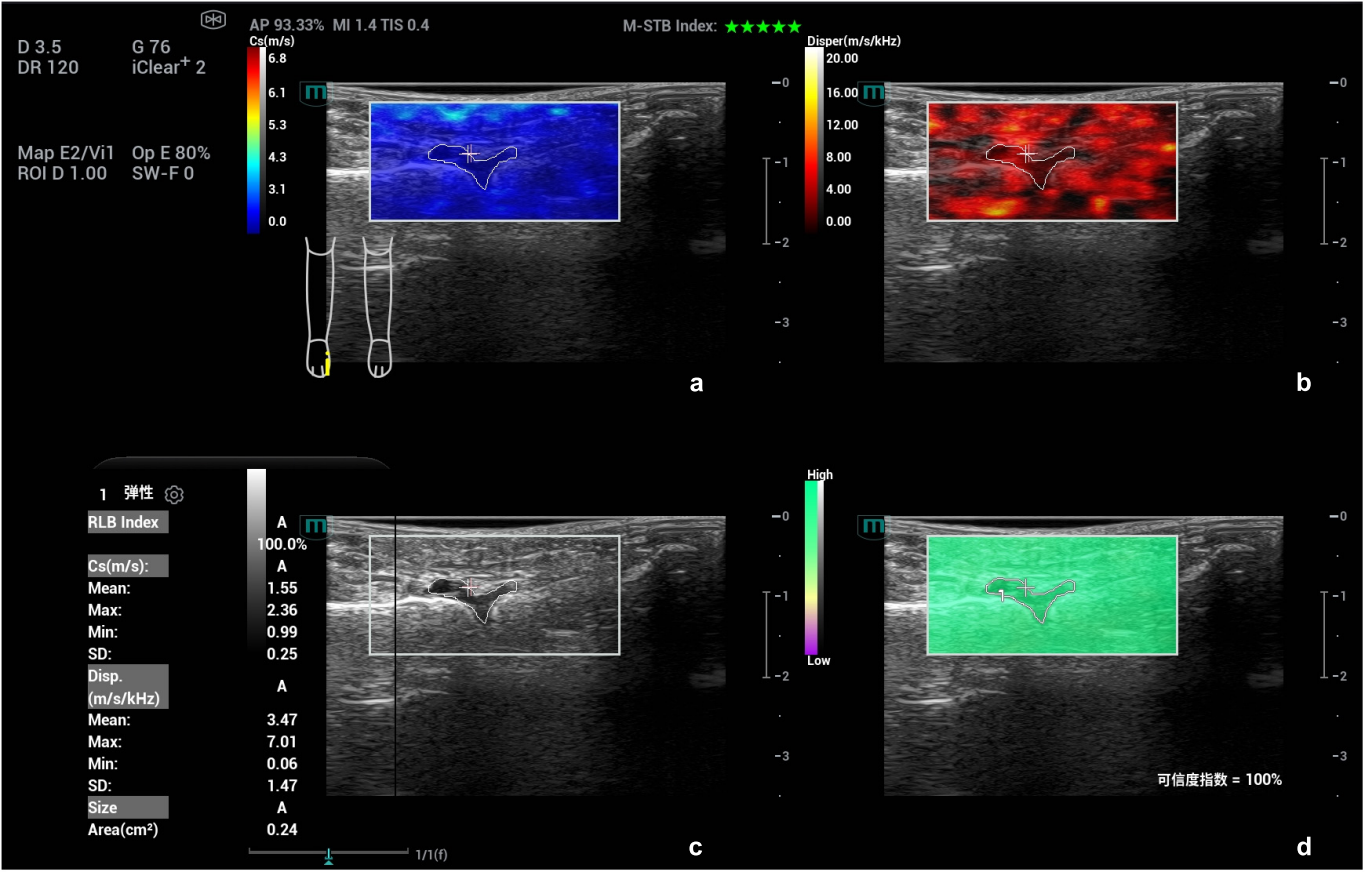
Schematic diagram of viscoelastic images (measurement of shear wave velocity and dispersion coefficient in the first metatarsophalangeal joint of the right foot). **(a)** (top left) Shear wave elasticity image; **(b)** (top right) Dispersion coefficient image; **(c)** (bottom left) Grayscale ultrasound image; **(d)** (bottom right) Reliability map (RLB).

Images were analyzed under the four-panel mode of viscoelastic imaging (Figures 2, 3). On the gray-scale ultrasound image, region of interest (ROI) was delineated along the edge of the thickened synovial, and the corresponding ROI was synchronously displayed on the shear wave elasticity and viscosity images. The system automatically acquired quantitative parameters: shear wave elastic parameters (reflecting tissue stiffness) including the mean, maximum, minimum, and standard deviation of shear wave velocity (C, m/s); viscosity imaging parameters (reflecting tissue viscosity) including the mean, maximum, minimum, and standard deviation of viscosity coefficient (V, Pa⋅s) and dispersion coefficient [D, (m/s)/kHz]. A total of 12 parameters were obtained. Image acquisition and data measurement were repeated 5 times, and the mean values were recorded and calculated.

To verify the reliability of ultrasound viscoelastic parameter measurement, a subset of images was selected for intra-rater and inter-rater reliability assessment. A total of 20 joint ultrasound images were randomly chosen, with 10 from the acute gout (AG) group and 10 from the non-AG group. Twelve viscoelastic parameters (including shear wave velocity, viscosity coefficient, and dispersion coefficient-related parameters) were independently and repeatedly measured by two physicians who had more than 5 years of professional experience in musculoskeletal ultrasound. The intraclass correlation coefficient (ICC) was adopted as the statistical indicator to evaluate intra-rater reliability and inter-rater reliability.

### 2.6 Statistical analysis

All statistical analyses were performed using SPSS 27.0 software (IBM Corp, Armonk, NY, USA) and MedCalc 22.0 software (MedCalc Software Ltd, Ostend, Belgium). Categorical data were expressed as *n* (%), and comparisons between groups were conducted using the chi-square (χ^2^) test. Continuous data were tested for normality via the Shapiro-Wilk test. Data conforming to a normal distribution were presented as $\bar{\text{X}}$ ± S, and comparisons between groups were performed using the independent samples *t*-test.Data with a skewed distribution were expressed as M (P_25_, P_75_), and comparisons between groups were conducted using the Mann-Whitney U test. For the assessment of measurement reliability (intra-rater and inter-rater reliability of viscoelastic parameters), the intraclass correlation coefficient (ICC) was used. With gout as the dependent variable and each quantitative parameter of ultrasound viscoelastic imaging as the independent variable, generalized estimating equations (GEE) was used to screen for independent influencing factors of gout among the ultrasound viscoelastic parameters. Receiver operating characteristic (ROC) curves were plotted to analyze the diagnostic efficacy of each parameter alone and in combination for acute gout. The DeLong test was used to compare the areas under the curves (AUC). A two-tailed *P*-value of less than 0.05 was considered to indicate statistically significant differences.

## 3 Results

### 3.1 Participant characteristics

A total of 45 patients (involving 70 joints) were enrolled in the gout group, aged 21∼78 years, with a mean age of (47.71 ± 13.43) years, including 38 males and 7 females. The non-gout group included 57 patients (involving 81 joints), aged 28∼75 years, with a mean age of (48.58 ± 15.05) years, including 42 males and 15 female. There were no statistically significant differences in the gender composition, age, and distribution of joint sizes between the two groups (χ^2^ = 1.721, *P* = 0.190; *t* = −0.303, *P* = 0.762; χ^2^ = 3.802, *P* = 0.149). The non-gout group consisted of 36 cases of rheumatoid arthritis, 12 case of osteoarthritis, 5 case of ankylosing spondylitis, 3 case of psoriatic arthritis, and 1 case of dermatomyositis. Comparisons of BMI, disease duration, and synovial thickness between the two groups showed no statistically significant differences (all *P* > 0.05). The serum uric acid level in the AG group was higher than that in the non-AG group, with a statistically significant difference (*t* = 10.249, *P* < 0.001) (Table 1).

**TABLE 1:** Baseline characteristics of patients.

Participant characteristics	AG group (*n* = 45)	Non-AG group (*n* = 57)	Statistical value	*P*-value
Age (years)	47.71 ± 13.43	48.58 ± 15.05	−0.303	0.762*
Male (case)	38 (84.44%)	42 (73.68%)	1.721	0.190^#^
BMI (kg/m^2^)	24.32 ± 1.39	23.97 ± 1.40	1.258	0.211*
Disease duration (months)	17.27 ± 9.82	20.54 ± 12.42	−1.446	0.151*
Serum uric acid (μmol/L)	455.18 ± 96.36	283.01 ± 65.74	10.249	<0.001*
Number of involved joints (case)	*n* = 70	*n* = 81	–	–
Large joints (hip joint, knee joint, shoulder joint)	15 (21.43%)	29 (35.80%)	3.802	0.149^#^
Medium joints (wrist joint, ankle joint, elbow joint)	21 (30.00%)	21 (25.93%)		
Small joints (hand joints, foot joints)	34 (48.57%)	31 (38.27%)		

AG, acute gout; NAG, non-acute gout; BMI, body mass index. *Independent samples *t*-test. #Chi-square(χ^2^).

### 3.2 Comparative of grayscale ultrasound images between the AG group and non-AG group

Statistical analysis was performed on the ultrasound signs between the AG and non-AG groups, including synovial thickness, joint effusion, bone erosion, double contour sign, and tophus. The results showed that there were no statistically significant differences in synovial thickness or bone erosion between the two groups (*P* > 0.05), while significant statistical differences were observed in joint effusion, double contour sign, and tophus between the two groups (*P* < 0.05). Using positive double contour sign or positive tophus as the diagnostic criterion, the sensitivity, specificity, AUC of ultrasound for diagnosing AG were 61.2%, 88.9%, and 0.752 (95% CI 0.670–0.833), respectively (Supplementary Table 1).

### 3.3 Reliability of viscoelastic parameter measurement

The results of reliability assessment showed that the intra-rater ICC of the 12 ultrasound viscoelastic parameters ranged from 0.83 to 0.91, and the inter-rater ICC ranged from 0.79 to 0.89. According to the commonly used ICC reliability evaluation criteria (ICC > 0.75 indicates good reliability), both intra-rater and inter-rater reliability of the parameter measurements in this study were classified as good, confirming the stability and credibility of the viscoelastic parameter measurement results (Supplementary Table 2).

### 3.4 Comparative of ultrasound viscoelastic parameters between the AG group and non-AG group

A total of 12 parameters were obtained in this study. Among them, the comparison of C_mean_, C_max_, C_min_, C_SD_, V_mean_, V_max_, V_SD_, D_mean_, D_max_, D_SD_ between the AG group and non-AG group showed statistically significant differences (all *P* < 0.05); while the differences in V_min_ and D_min_ between the two groups were not statistically significant (*P* > 0.05) (Table 2).

**TABLE 2:** Comparison of ultrasound viscoelastic parameters between AG group and non-AG group.

Parameters	AG group	Non-AG group	Statistical value	*P*-value
Number of involved joints (case)	70	81	–	–
C_mean_ (m/s)	2.69 (2.44, 3.08)	2.13 (1.78, 2.49)	−7.103	<0.001^▲^
C_max_ (m/s)	4.43 (3.83, 5.37)	3.29 (2.77, 4.06)	−5.685	<0.001^▲^
C_min_ (m/s)	1.32 ± 0.54	1.08 ± 0.58	2.542	0.012*
C_SD_ (m/s)	0.59 (0.45, 0.80)	0.45 (0.29, 0.68)	−3.030	0.002^▲^
V_mean_ (Pa.s)	0.88 (0.50, 1.30)	0.64 (0.43, 0.80)	−2.965	0.003^▲^
V_max_ (Pa.s)	3.18 (1.76, 4.59)	1.79 (1.30, 3.16)	−3.364	0.001^▲^
V_min_ (Pa.s)	0.00 (0.00, 0.00)	0.00 (0.00, 0.00)	−0.260	0.795^▲^
V_SD_ (Pa.s)	0.64 (0.41, 1.01)	0.39 (0.27, 0.61)	−4.079	<0.001^▲^
D_mean_ [(m/s)/kHz]	5.43 (4.05, 6.64)	4.09 (3.01, 6.36)	−2.493	0.013^▲^
D_max_ [(m/s)/kHz]	18.12 (10.68, 20.00)	10.91 (8.09, 15.05)	−3.960	<0.001^▲^
D_min_ [(m/s)/kHz]	0.00 (0.00, 1.15)	0.00 (0.00, 1.40)	−0.416	0.677^▲^
D_SD_ [(m/s)/kHz]	3.17 (1.97, 3.81)	1.95 (1.51, 2.80)	−3.931	<0.001^▲^

AG, acute gout; NAG, non-acute gout; C, shear wave velocity; V, viscosity coefficient; D, dispersion coefficient. ^▲^Mann-Whitney U test. *Independent samples *t*-test.

### 3.5 Generalized estimating equations (GEE) analysis of ultrasound viscoelastic parameters for predicting AG

With gout as the dependent variable and the ultrasound viscoelastic parameter (C_mean_, C_max_, C_min_, C_SD_, V_mean_, V_max_, V_SD_, D_mean_, D_max_, D_SD_) which showed statistically significant differences between the AG group and non-AG group as the independent variables, GEE analysis was performed. The results showed that C_mean_ and D_max_ were independent risk factors for predicting AG (both OR > 1, *P* < 0.05). Based on the results of the GEE analysis, a multi-parameter combined diagnostic equation was established as follows: Logit(P) = (−11.186) + (3.456 × C_mean_) + (0.191 × D_max_) (Table 3).

**TABLE 3:** Generalized estimating equations (GEE) analysis results of ultrasound viscoelastic parameters for predicting acute gout (AG).

Parameters	B	SE.	Wald	*P*	OR	95% CI for OR	
						Lower	Upper
C_mean_	3.456	0.728	22.550	<0.001	31.683	7.610	131.912
D_max_	0.191	0.048	15.686	<0.001	1.210	1.101	1.330
Constant	−11.186	1.833	37.231	<0.001	0.000	–	–

C, shear wave velocity; D, dispersion coefficient; B, unstandardized regression coefficient; SE, standard error; OR, odds ratio.

### 3.6 Exploration of optimal ultrasound viscoelastic parameters

**FIGURE 4:**
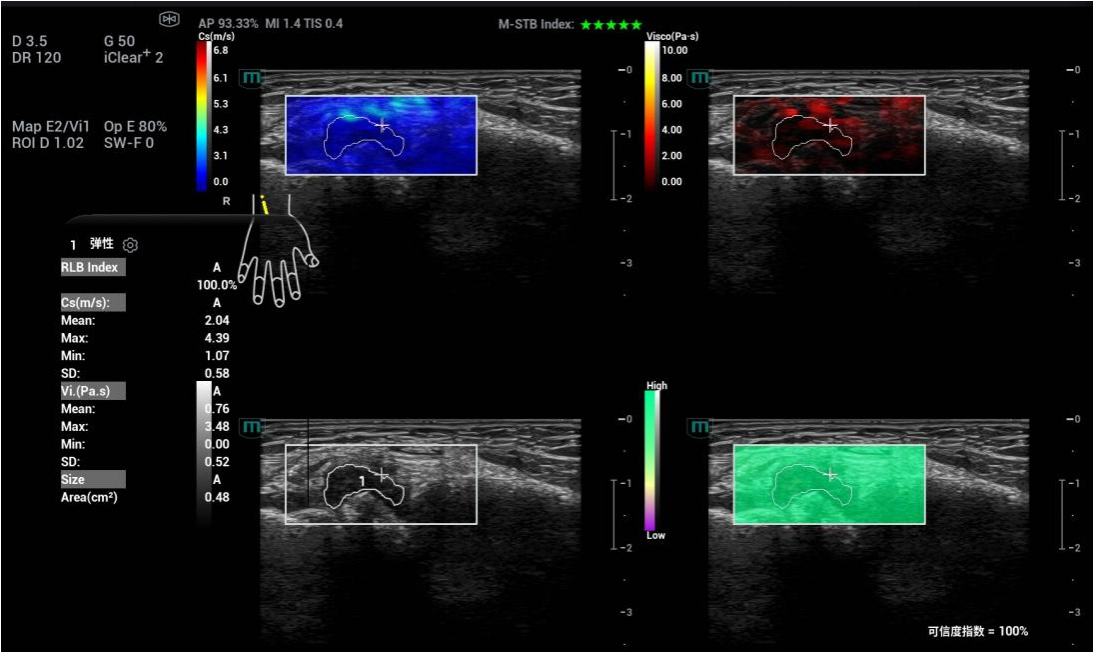
Ultrasound viscoelastic image of the left elbow joint in a patient with acute gout (AG). A 69-year-old male patient. Ultrasound viscoelastic imaging showed that the thickened synovium of the left elbow joint had a Cmean of 2.98 m/s (>cut-off value of 2.36 m/s) and a Dmean of 8.47 (m/s)/kHz [>cutoff value of 4.35 (m/s)/kHz].

**FIGURE 5:**
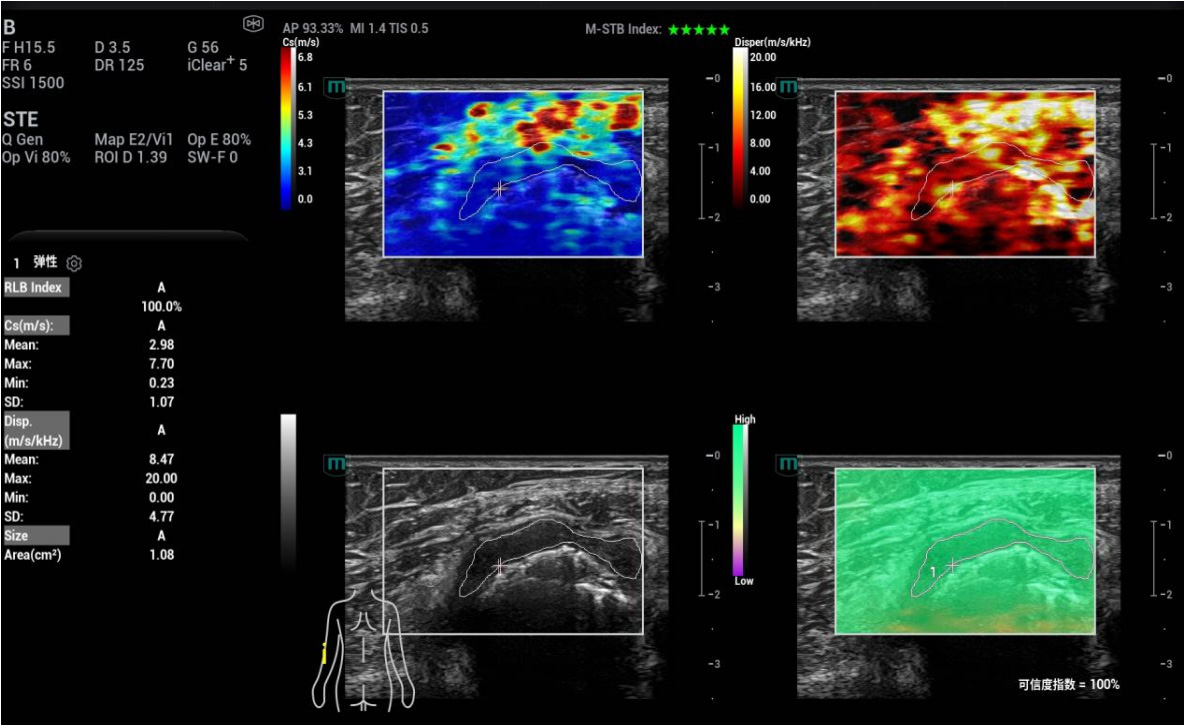
Ultrasound viscoelastic image of the right wrist joint in a patient with rheumatoid arthritis. A 60-year-old male patient. Ultrasound viscoelastic imaging showed that the thickened synovium of the right wrist joint had a Cmean of 2.04 m/s (<cutoff value of 2.36 m/s) and a Vmean of 0.76 Pa⋅s (<cut-off value of 0.81 Pa⋅s).

Among the single parameters, C_mean_ showed the highest diagnostic efficacy, with an AUC of 0.836 (95% CI 0.774∼0.897). The optimal cut-off value was 2.36 m/s (Figures 4, 5), at which the sensitivity and specificity for diagnosing AG were 81.4% and 71.6%, respectively. The AUC of the GEE model was 0.885 (95% CI 0.833∼0.938), with an optimal cutoff value of 0.41, and the corresponding sensitivity and, specificity for diagnosing AG were 87.1% and 76.5%, respectively (Table 4).

**TABLE 4:** ROC curve analysis of ultrasound viscoelastic parameters for diagnosing AG.

Parameters	AUC	95% CI	*P*	Cut of value	Sensitivity	Specificity	Youden index
GEE model	0.885	0.833∼0.938	<0.001	0.41	0.871	0.765	0.636
C_mean_	0.836	0.774∼0.897	<0.001	2.36 m/s	0.814	0.716	0.530
C_max_	0.769	0.693∼0.844	<0.001	3.85 m/s	0.757	0.704	0.461
C_min_	0.620	0.530∼0.710	0.011	1.22 m/s	0.643	0.630	0.273
C_SD_	0.643	0.556∼0.731	0.002	0.43 m/s	0.814	0.481	0.295
V_mean_	0.640	0.549∼0.731	0.003	0.81 Pa.s	0.557	0.765	0.322
V_max_	0.659	0.572∼0.746	0.001	2.95 Pa.s	0.543	0.753	0.296
V_SD_	0.693	0.608∼0.777	<0.001	0.55 Pa.s	0.643	0.679	0.322
D_mean_	0.618	0.528∼0.708	0.013	4.35 (m/s)/kHz	0.729	0.556	0.285
D_max_	0.686	0.599∼0.774	<0.001	15.75 (m/s)/kHz	0.629	0.778	0.407
D_SD_	0.686	0.598∼0.773	<0.001	2.82 (m/s)/kHz	0.600	0.790	0.390

C, shear wave velocity; V, viscosity coefficient; D, dispersion coefficient; GEE, generalized estimating equations.

**FIGURE 6:**
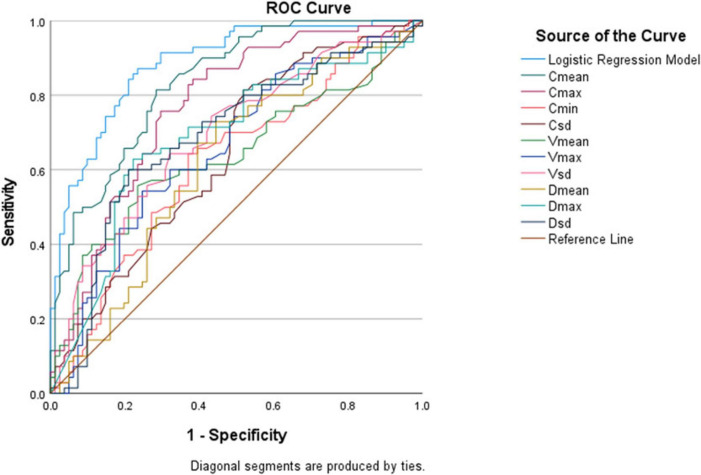
Receiver operating characteristic (ROC) curves of various ultrasound viscoelastic parameters for diagnosing acute acute gout (AG).

The DeLong test was used to compare the AUC values of C_mean_ and the GEE model. The results showed that the difference between the two was statistically significant (*Z* = 2.130, *P* = 0.033). The GEE model had the largest AUC value, indicating that compared with a single parameter, the combination of multiple viscoelastic parameters has better predictive value for acute AG (Figure 6).

## 4 Discussion

Acute gout (AG) is an acute inflammatory response caused by the deposition of MSU crystals in joints and surrounding soft tissues. Early diagnosis of gout is a key link in improving patients’ prognosis, which can significantly reduce the rate of deformity and improve quality of life (19). Currently, specific ultrasound signs have high specificity in chronic gout but still have certain limitations in the assessment of AG. Therefore, exploring emerging ultrasound diagnostic technologies is of great significance for improving the early diagnosis level of AG. In this study, a novel viscoelastic biomechanical model was used to quantitatively evaluate the elastic and viscous physical characteristics of joint synovium. This study explored whether viscoelastic parameters have certain value in the diagnosis of AG in patients with acute joint swelling and pain.

The advantage of ultrasonic viscoelastic imaging technology lies in its ability to simultaneously measure elastic parameters (including shear wave velocity) and viscous parameters (including dispersion coefficient and viscosity coefficient), which is more consistent with the mechanical properties of human tissues. Additionally, it can provide multi-parameter quantitative assessment and has the advantages of non-invasiveness, low cost, and high sensitivity. Currently, several studies have confirmed the application value of shear wave elastography in musculoskeletal ultrasound (20–25).

Compared with grayscale ultrasound, the additional value of viscoelastic imaging lies in the following aspects: it quantifies the mechanical properties of tissues (elasticity and viscosity), making up for the subjectivity of qualitative assessment by grayscale ultrasound; and it improves the diagnostic sensitivity for acute gout. Specifically, the diagnostic sensitivity of the viscoelastic parameter prediction model (87.1%) is significantly higher than that of grayscale ultrasound (61.2%), and its area under the receiver operating characteristic curve (AUC = 0.885) is also higher than that of grayscale ultrasound (AUC = 0.752).

The inter-group difference analysis in this study showed that all shear wave velocity parameters (C_mean_, C_max_, C_min_, C_SD_) in the AG group were higher than those in the non-AG group (*P* < 0.05 for all). This may be due to the deposition of MSU crystals increasing the stiffness of the synovium, leading to an increase in shear wave velocity. Moreover, the joint lesions in the AG group were more heterogeneous, resulting in a higher C_SD_ than in the non-AG group. This is consistent with the trend observed in the study by Tang et al. (26), but there are differences in the measurement method of the ROI for elasticity image between our study and that of Tang Y. The previous study adopted a circular fixed-diameter method for ROI selection, while we used a free-hand tracing method to include the entire lesion area in the ROI. Furthermore, in tour study, shear wave velocity (C) was used to reflect tissue elasticity, rather than elastic modulus, which is also a difference from previous studies. This is because Zhu et al. (27) proposed that due to the anisotropy of musculoskeletal ultrasound, shear wave velocity is recommended for elasticity evaluation.

Previous viscoelastic imaging studies (28–30) on mice and patients with hepatitis have shown that when tissues undergo inflammatory changes, tissue viscosity increases, and there are differences in shear wave velocity and dispersion coefficient measured at different inflammatory stages. It was further concluded that shear wave velocity and viscous parameters are mainly related to liver fibrosis grading and inflammatory necrosis grading. Studies have also found that elastic parameters are superior to viscosity parameters in evaluating liver fibrosis, while viscosity parameters are superior to elastic parameters in evaluating the degree of inflammation and necrosis (31, 32). A multicenter prospective study conducted by Jia et al. (5) indicated that malignant breast lesions are more viscous than benign lesions.

Viscosity coefficient and dispersion coefficient have not been applied in joint synovium in previous studies, and this study conducted a preliminary exploration. We found that the viscous parameters (V_mean_, V_max_, V_SD_) in the AG group were all higher than those in the non-AG group (*P* < 0.05 for all), while there was no significant difference in V_min_ between the two groups (*P* = 0.795). This may be due to the activation of innate immunity by MSU crystals in AG, triggering a severe inflammatory response of the synovium. MSU activates the positive feedback mechanism mediated by mononuclear macrophages and neutrophils, and the main histological features of this process are hyperplasia of synovial lining cells and infiltration of neutrophils, mononuclear macrophages, and lymphocytes (33, 34). Unlike chronic arthritis such as rheumatoid arthritis and psoriasis, AG has a rapid onset and peaks quickly, which is pathogenetically different from these chronic inflammatory diseases. These factors may collectively lead to an increase in tissue viscosity in AG. Previously, Li W’s (14) study suggested that the viscosity coefficient is sensitive to mucin-rich or necrotic tissues.

In addition, the dispersion parameters (D_mean_, D_max_, D_SD_) in the AG group were higher than those in the non-AG group (*P* < 0.05 for all), reflecting that the structure of synovial hyperplasia in AG is more complex. Due to the deposition of urate crystals, the internal structure of the synovium becomes more complex, and the dispersion slope can reflect tissue heterogeneity. In previous studies on viscoelastic imaging, dispersion parameters were mostly used for evaluating liver lesions (35–38), and the findings showed that the dispersion slope is more sensitive to liver fibrosis and structurally disordered tissues. The inter-group comparison results in this study showed that both elastic and viscous parameters can effectively reflect the tissue characteristics of the synovium in the joints, providing an important quantitative basis for the diagnosis of AG.

In this study, GEE was used to screen out C_mean_ and D_max_ as statistically significant predictors of AG. In the construction and evaluation of the prediction model, compared with the single-parameter model, the combined viscoelastic parameter model showed stronger predictive ability. Among the comparisons of single-parameter models, the C_mean_ univariate model had the best predictive performance, with an AUC of 0.836(95% CI 0.774∼0.897)and an optimal cut-off value of 2.36 m/s. Previously, a study by Wang Q(21) suggested that ultrasound shear wave elastography (SWE) has high diagnostic performance in differentiating between the acute phase and intercritical phase of gout, with the AUC of mean elastic modulus (E_mean_) being 0.882, which is consistent with the trend of the results in this study. The AUC of the combined parameters model was 0.885(95%CI 0.833∼0.938), which was higher than that of the univariate model (*Z* = 2.130, *P* = 0.033). This indicates that the combined viscoelastic parameters model can more effectively assist in the diagnosis of AG.

This study has certain limitations. First, as a single-center exploratory study, it has a relatively small sample size and no stratification by joint size (large joints, medium joints, and small joints), making it difficult to clarify the specific application value of this technology in joints of different sizes; future large-sample, multi-center studies are needed for further verification. Second, although the initially established diagnostic cut-off values of ultrasound viscoelastic parameters show good discriminative effects in internal data, their generalizability and clinical applicability still require external validation in prospective, multi-center independent cohorts with more diverse population characteristics. Finally, the study did not perform Type I error correction for multiple comparisons, which may overestimate the statistical significance of some parameters and pose potential false-positive risks; in the future, the reliability of results will be improved by expanding the sample size and focusing on the validation of core parameters (e.g., C_mean_ and D_max_ screened by GEE analysis).

## 5 Conclusion

In summary, our research findings indicate that ultrasonic viscoelastic imaging technology has certain diagnostic value in the non-invasive diagnosis of AG. The diagnostic efficacy of the combined multi-parameter model is superior to that of single-parameter diagnosis. It should be noted that although ultrasound viscoelastic imaging demonstrates strong diagnostic efficacy, we do not recommend using viscoelastic imaging as the sole examination method, but rather as an auxiliary means for conventional ultrasound.

## Data Availability

The raw data supporting the conclusions of this article will be made available by the authors, without undue reservation.
