# Supplementary material for: Exploration of acute gout diagnosis based on ultrasound viscoelastic imaging: quantitative parameter analysis and clinical validation
Source: Front Med (Lausanne). 2025 Dec 17;12:1729517. doi: 10.3389/fmed.2025.1729517 (PMC12753903; doi:10.3389/fmed.2025.1729517)
Supplement: Supplementary file 1 [file Table_1.docx]

Supplementary Material

# Supplementary Tables 1

Supplementary table 1. Comparison of grayscale ultrasound ultrasound images between AG group and non-AG group

| Grayscale ultrasound sign | GA group | Non-GA group | Statistical value | *P*-value |
| --- | --- | --- | --- | --- |
| Number of involved joints (case) | 70 | 81 | - | - |
| Synovial thickness(mm) | 3.10(2.60, 4.55) | 3.90(2.35, 5.20) | -1.052 | 0.293^▲^ |
| joint effusion | 36（51.42%） | 25（30.86%） | 6.595 | 0.010^#^ |
| bone erosion | 8（11.42%） | 13（16.04%） | 0.670 | 0.413^#^ |
| double contour sign | 30（42.85%） | 6 （7.40%） | 25.989 | < 0.001^#^ |
| tophus | 28（40.00%） | 4 （4.93%） | 25.580 | < 0.001^#^ |

AG: Acute Gout, NAG: Non Acute Gout ,▲: Mann-Whitney U test, #: chi-square(*χ*²)
