# Supplementary material for: Exploration of acute gout diagnosis based on ultrasound viscoelastic imaging: quantitative parameter analysis and clinical validation
Source: Front Med (Lausanne). 2025 Dec 17;12:1729517. doi: 10.3389/fmed.2025.1729517 (PMC12753903; doi:10.3389/fmed.2025.1729517)
Supplement: Supplementary file 2 [file Table_2.docx]

Supplementary Material

# Supplementary Table 2

Supplementary table 2. Intra- and inter-rater reliability of ultrasound viscoelastic parameters

| Parameters | Inter-rater Reliability (ICC) | 95% CI for Inter-rater ICC | Intra-rater Reliability (ICC) | 95% CI for Intra-rater ICC |
| --- | --- | --- | --- | --- |
| C_mean_ (m/s) | 0.828 | 0.616~0.928 | 0.891 | 0.746~0.955 |
| C_max_ (m/s) | 0.798 | 0.558~0.915 | 0.852 | 0.664~0.939 |
| C_min_ (m/s) | 0.864 | 0.689~0.944 | 0.911 | 0.789~0.964 |
| C_SD_ (m/s) | 0.837 | 0.634~0.932 | 0.866 | 0.692~0.945 |
| V_mean_ (Pa.s) | 0.863 | 0.687~0.944 | 0.869 | 0.699~0.946 |
| V_max_ (Pa.s) | 0.898 | 0.762~0.958 | 0.913 | 0.794~0.965 |
| V_min_ (Pa.s) | 0.865 | 0.690~0.944 | 0.883 | 0.729~0.952 |
| V_SD_ (Pa.s) | 0.885 | 0.734~0.953 | 0.900 | 0.765~0.959 |
| D_mean_[(m/s)/kHz] | 0.791 | 0.544~0.912 | 0.884 | 0.721~0.953 |
| D_max_[(m/s)/kHz] | 0.856 | 0.673~0.940 | 0.905 | 0.777~0.961 |
| D_min_[(m/s)/kHz] | 0.869 | 0.699~0.946 | 0.871 | 0.704~0.947 |
| D_SD_ [(m/s)/kHz] | 0.790 | 0.543~0.911 | 0.836 | 0.631~0.932 |

C: Shear Wave Velocity, V: Viscosity Coefficient, D:Dispersion Coefficient, ICC:
